# Interphase investigation of modified McLachlan model and the 3D finite element method for electrical conductivity

**DOI:** 10.1016/j.heliyon.2024.e41621

**Published:** 2025-01-01

**Authors:** Muhammad Zulkarnain, Irianto Harny, A.A.M. Damanhuri

**Affiliations:** a Faculty of Mechanical Technology and Engineering, Universiti Teknikal Malaysia Melaka (UTeM), Durian Tunggal, Melaka, 76100, Malaysia; b Department General Education, Faculty of Resilience, Rabdan Academy, Abu Dhabi, 22401, United Arab Emirates

**Keywords:** Percolation, Conductivity, Interphase, Tunneling, Modeling

## Abstract

This paper explores the electrical conductivity interphase of Ag/Epoxy composite using modified McLachlan theory and 3D finite element composite model through experimental verification. The model characteristic presents conductivity as a dynamic function influenced by particle content, particle electrical properties, electrical properties transition, and an exponent. This model was meticulously crafted, considering the intricate interplay between the polymer matrix and silver particles, the tunnelling distance between adjacent silver particles, and the interphase regions around particles. This model has proven its mettle through rigorous analysis of experimental results and the impact of various parameters on conductivity. The predictions have shown impressive alignment with the experimental data, highlighting the crucial roles played by the parameters in the conductivity of silver composites where the percolation threshold reached 6 vol % of filler loading. The experimental study demonstrated that the electrical conductivity was 3.84 × 10^−1^ S/cm for micro-sized particles and 1.32 × 10^−2^ S/cm for nano-sized particles. Notably, a large tunnelling distance drastically reduces conductivity, while higher and slighter surface energies of the polymer matrix and filler enhance conductivity. Furthermore, a thin interphase yields minimal conductivity, whereas a thick interphase and low waviness improve conductivity. The McLachlan-modified model falls slightly short in accuracy compared to the 3D finite element method models. Adjustments to the equations can enhance its alignment with experimental data.

## 1 Introduction

Silver particles possess remarkable electrical conductivity which is used in composite for several applications. Previous studies have delved into the correlation between total cure shrinkage and the conductivity of composite, revealing that higher shrinkage leads to lower resistance or improved conductivity of the materials [1,2]. These advanced materials offer significant advantages for high implementation possibilities in electronics electromagnetic shielding, cathodic protection systems, structural health monitoring (SHM) and sensors. Consequently, it was vital to optimize conductivity by adjusting the material structure and processing characteristics for particle composites. The conductivity of particle composites originates from the transition phase known as the percolation threshold, where interconnecting networks pattern. The early history of the application of electrically conductive adhesive has been patented using thermoset polymer with Silver (Ag) filler, leading to extensive research into conductive adhesives for die manufacturing and terminal bonding of integrated circuits [3]. Notably, high-purity fine Ag particles serve as conductive paste and adhesive additives for electrodes and electrical parts. Nanometer-sized colloids of Ag particles have proven to be effective materials for producing conductive and transparent thin films. Among electrically conductive adhesive (ECA) composites, Ag particles stand out as the most widely used due to their lower resistivity, ease of processing, and stability in various environmental conditions [[4], [5], [6], [7], [8]].

A composite of Ag/epoxy has been developed to improve large-pitch interconnections at room temperature and address challenges in fine-pitch interconnections for miniaturized devices. While the conductive adhesives have limitations, efforts are focused on enhancing electrical properties, contact resistance performance and impact ability through refined designs and formulations. The morphological analysis of ECAs is crucial for understanding their electric transport properties, particularly near the percolation threshold transition. This study aims to optimize particle structure design to improve electrical conductivity. Scanning electron microscope (SEM) micrographs are used to study particle distribution patterns and performance in the polymeric system [9]. This analysis provides valuable input for the interconnection of ECAs, it is essential to achieve exceptional electrical, thermal and mechanical performance to ensure long-term service reliability. Previous researchers revised the morphology by incorporating silver flake content has demonstrated a substantial impact on both the electrical and mechanical performance of the ECAs. The strategic alignment of Ag flakes within the cured composite, coupled with their interaction with adhesive chains extracted from tetrafunctional polyurethane acrylate oligomer, serves to greatly elevate the material, and enhance its electrical conductivity and adhesion properties material [10]. Morphological changes were observed during the oxidation of Cu particles within the matrix. Although the percolation threshold was achieved, resulting in the pattern of a particle connecting with conductivity, unfortunately, the resistivity increased. The enhancement of Cu particles through Ag coating successfully increased the conductivity, as confirmed by morphological analysis and reduced resistance [11].

The research in this field has explored various parameters to enhance the percolation threshold of ECA. Many experts have made valuable recommendations for improving ECA properties, such as optimizing particle distribution and reducing particle contact resistance [[12], [13], [14], [15], [16]]. According to the literature, the most compelling topics revolve around the impact of filler concentration (volume fraction), particle morphology, particle size distribution, particle shape, particle surface and interaction, and adhesive pre-treatment on the electrical properties of ECAs. The upcoming subsections will delve into factors that influence silver-filled composites, including particle shape and size. The conductivity of a material can be enhanced through networking, which involves promoting the formation of a particle connecting by bringing particles into contact. On the other hand, the network path increases harness electrical charge by utilizing the conductor properties of the tunnelling effect in the absence of direct interconnecting between the particles, achieved through electric charge based on neighbouring distance [[17], [18], [19]]. For material designers, predicting percolation threshold is crucial in developing polymer nanocomposites, as it allows for the maximization of effective electrical conductivity with the presented content of particles. Consequently, numerous researches have focused on properly adding and dispersion of great particles in electrical properties [[20], [21], [22], [23]]. The resulting composite demonstrated outstanding characteristics by enhancing the dispersibility of particles, especially in facilitating the tunnelling effect for the networking path. Applying polysilazane (PSZ) to the Cu particle surface led to remarkable thermal conductivity through uniform particle distribution and the development of effective heat-connecting networks [24]. Morphology controlling the shape and structure of particles is crucial for optimizing both thermal and electrical performance in composite materials. Applying ceramic coatings to particles such as metals of Ag and Cu, can ensure even distribution within the polymer matrix, forming a continuous network that efficiently facilitates heat and electricity transmission [25]. Other research holds equal significance, including biodegradable and biocompatible polymer nanocomposites are a promising area of research with significant applications in biomedical devices, tissue engineering, and sustainable materials. Conductivity studies in these materials are crucial for functionalities in biosensors, drug delivery systems, and bioelectronics [[26], [27], [28], [29]]. The investigation focuses on developing and characterizing eco-friendly Magnesium Oxide (MgO) nanofiller-reinforced hydroxypropyl methylcellulose (HPMC) polymer composites tailored for electronic applications has been done [30]. The MgO-HPMC nanocomposites are not just innovative; they are essential for advancing eco-friendly electronics, and significantly reducing electronic waste while promoting environmental sustainability. However, it's important to note that dielectric loss decreases with higher frequency and MgO concentration. This area of research integrates material science, bioengineering, and nanotechnology, holding the potential to revolutionize both eco-friendly electronics and the future of medical devices. Investing in this research is not only a step toward innovation but also a commitment to a sustainable future.

Previous researchers have delved into the tunnelling of electrical transport in nanoparticle networks, with many employing traditional equations to estimate the potential of polymer nanocomposites with an understanding of electrical properties transition and conductivity characteristics. Various straightforward models have emerged to predict the performance of nanoparticles in electrical conductivity, drawing on micromechanics to predict conductivity based on nano-particles and particle network properties, such as particle array setting, tunnelling distance, and particle agglomeration. Nevertheless, the complex and ambiguous terms they employ restrict their practical application [[30], [31], [32], [33], [34]]. Moreover, prevailing models frequently overlook the impact of interphase zones on the insulator-to-conductor transition phase level of nanocomposites. The substantial unlocking potential of nanoparticles lies in their impressive surface area per unit volume, bolstered by the robust interfacial interaction between the polymer matrix and nanoparticle. It gives rise to distinct interphase regions within nanocomposites. These regions play an important role in governing the mechanical behaviour of nanocomposites, contributing to their reinforcement. Additionally, the interphase regions near nanoparticles have the high possibility to perform extensively in nanoparticle composite channels even before the nanoparticles are fully connected, thus reducing the percolation threshold. While previous research has focused on the influence of interphase threshold conductivity on the mechanical characteristics of nanoparticle composites, the influence of interphase regions on the electrical properties of nanoparticle composites has been largely overlooked. The study employed various analytical methods to focus on interphase percolation. The study comparison of particles by using soft and hard-core methods for modelling percolation threshold sheds light on the effectiveness of different techniques. By utilizing Monte Carlo (MC) simulation, the study discovered the fascinating connection between PT and eliminating the volume in both methods [35,36]. Proposed improvements of established analytical models, such as Mori-Tanaka, Balberg, Maxwell's, and McLachlan, hold promise for application in nanocomposites [[37], [38], [39], [40], [41], [42]]. These adjustments consider crucial parameters including several parameters that are considered in relationship to the interphase parameter, the aspect ratio of the filler, and the surface energy between the filler and matrix are crucial considerations. Notably, these modifications have yielded successful predictions consistent with test results, demonstrating their potential. Among the various parameters, the interphase between fibres and the matrix emerges as pivotal in influencing tensile modulus, strength, and electrical conductivity. Understanding the interphase zone between the macromolecular chains and the fillers is crucial. When lower concentrations of nanofillers are used, the structure of nanocomposites is well distributed, and the interaction between the nanoparticle and bulk polymer effectively creates the interphase region. However, as the concentration of nanofillers is added, the volume content of the nanoparticles also increases, leading to an interconnected interphase. This happens because the nanofillers cannot disperse well in the polymer matrix. The agglomeration of nanofillers reduces their surface area and causes the interphase to overlap. This overlap may enable the movement of electrons to reach the electrode faster due to shorter distances between nanoparticles.

The interphase undeniably plays a crucial role in determining the characteristics and properties of a composite material. One of its most important functions is influencing the physical properties of the material. Previous researchers have indicated that the formation of interphases between particles and polymers can potentially improve the uniformity of the polymer system and prevent phase separation in composite structures, especially in a mixture of two immiscible polymers [43]. The interphase regions positively impact nanoparticle percolation in polymer nanoparticle composites by creating a continuous connecting pattern, establishing the percolation threshold before full nanoparticle connection, which affects the transition conductivity from insulator to conductor before the real connection of nanoparticles occurs [44]. This paper investigates the interphase for electrical conductivity of Ag/Epoxy composite using the modified McLachlan theory, compared to the 3D finite element composite model through experimental verification data. The study focused on composites with two components (filler and matrix) and interphase region.

This paper introduces a sophisticated 3D model that significantly enhances our understanding of composite conductivity by examining crucial factors such as interfacial tension between the polymer matrix and nano- and micro-particles, the tunnelling distance between adjacent particles, and the interphase region surrounding these particles. By effectively incorporating these essential parameters, the model not only predicts conductivity with precision but also offers valuable insights into the mechanics of the composites. The model's reliability is demonstrated through its alignment with experimental conductivity data from various polymer-silver (Ag) composites, further supplemented by an analysis of how these key parameters influence conductivity in comparison to McLachlan's modified analysis. Integrating FEM-based agglomeration modelling with modified McLachlan theory presents a powerful opportunity to enhance composite materials. The Finite Element Method (FEM) is essential for improving McLachlan's predictions, as it generates crucial data on filler fraction and interparticle distance for his equations. Moreover, the percolation thresholds from McLachlan's modified theory validate and refine FEM models, boosting their accuracy. By merging insights from FEM with McLachlan's modified comprehensive properties, we can effectively connect microstructural behaviours with macroscopic performance. This unified approach not only drives material optimization but also fosters groundbreaking innovations in composite materials.

To advance our understanding of material behaviour and optimize performance, precise simulations are essential. The Finite Element Method (FEM) breaks down complex materials into manageable sections called finite elements, where equations governing particle interactions, like van der Waals forces, are solved. This method effectively handles the geometries and boundary conditions of agglomerated particles using sophisticated meshing techniques. FEM simulations incorporate critical parameters such as particle size, shape, density, and inter-particle forces, accurately modelling the dynamic processes of agglomeration. It assesses how particles cluster and their impact on macroscopic properties like conductivity, evaluating factors such as tunnelling distances, contact resistance, and percolation thresholds to determine agglomerates' effects on conductivity. This framework supports adjustments in particle properties and material composition to identify optimal configurations for specific applications. Validating the model's predictions against experimental data ensures reliability, with any discrepancies used for refinement.

## 2 Experimental method details

### 2.1 Particles and adhesive materials

The adhesive used in this research was epoxy-type EPON™ introduced as Resin 8281 with a market named EPON 8281, it was sourced from the United States of America and produced by Hexion company. The adhesive curing cycle uses temperature treatment in the oven by a Polyetheramine (PEA) agent supplied by BASF company. The adhesive used was one part (100) mixed to one-third (32) of the agent ratio for pre-curing before temperature treatment in the oven. Several particles were added to the adhesive and mixed before the agent affected the curing cycle. Two types of particle sizes were selected in this study, particle size for 80 nm which represents nano-particles while an average of 2–3.5 μm represents micro-sized. The chloroform lubricant particle was required to reduce agglomeration before being mixed with an adhesive. While a particle size analyzer using Zetasizer Malvern, based on dynamic light scattering (DLS) and electrophoretic light scattering (ELS), is ideal for nanoparticles and colloids in suspension.

### 2.2 Conductive adhesive procedure

**Fig. 1:**
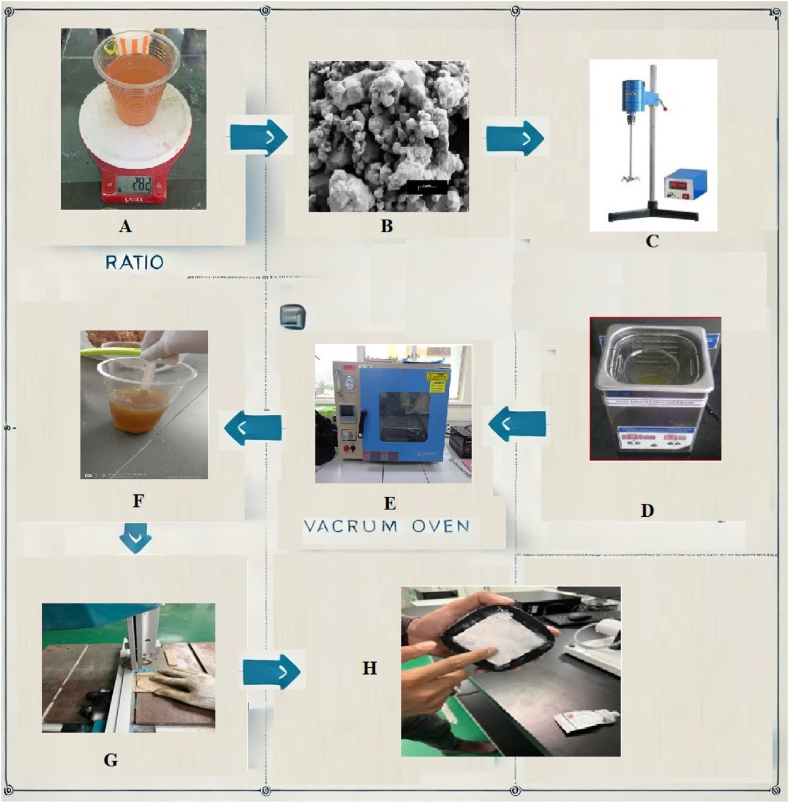
Composite preparation for electrical performance; A. Ratio measurement, B. Ag Particle, C. Stirrer processing, D. Sonication bath processing, E. Vacuum oven processing, F. Hardener mixing, G. Sample cutting, H. Sample testing.

Fig. 1 illustrates the comprehensive preparation of materials for micro- and nano-composites. The resin was thoughtfully formulated across a range of 0–8 vol%, as shown in Fig. 1(A), laying a solid foundation for electrical performance applications. A conductive adhesive was meticulously prepared using a varied volume of fillers in the range of 2 %, 4 %, 6 % and 8 % to ensure the formation of a strong particle network to perform the percolation threshold (Fig. 1B). After stirring for 10 min (Fig. 1C), the adhesive and particle to considered the physical blend, and then the blending was vibrated at a low frequency using a sonification bath for 30 min to achieve optimal particle distribution (Fig. 1D). Moreover, vacuuming the blending for 0.5 h at 35 °C helped eliminate bubbles (Fig. 1E). The addition of 32 parts by weight of curing agent to 100 parts of EPON 8281 was followed by a 10-min stirring period (Fig. 1F). To complete the process, the blending was cured by using temperature treatment in the oven for 2 h at room temperature and then heating the sample at 100 °C take the processing in 1 h, and finally, post-curing at 125 °C to complete in 3 h. Before conducting the sample testing, it meticulously prepared the process of cutting the composite sample with a bandsaw. This crucial step was followed by thorough electrical testing, clearly depicted in Fig. 1G and H. Ensuring precision in these procedures is vital for obtaining reliable data.

### 2.3 Electrical conductivity test

The ECA samples demonstrated strong electrical conductivities, indicating their high performance, the Instek LCR-817 device was used in our assessments. The sample was prepared in size of 2 cm × 2 cm by applying 1 V to transfer the current.

Discover the power of electrical conductivity (*σ*) - the key to unlocking a material's ability to conduct electricity. By following Ohm's Law, this study can precisely determine this remarkable property. This testing serves the vital purpose of determining the composite's conductivity by measuring the volume resistance, *Rv*.

To understand and apply the electrical resistance concept, volume resistivity (*ρ*_*v*_) is a critical measure that is essential for developing reliable and high-performing electrical materials. When an ohm centimetre is applied, it represents the electrical resistance across a material occupying a volume of one cubic centimetre. The volume resistance is accurately determined by measuring the voltage potential (*∅*) through the material's opposite side and measuring the magnitude of the resultant current (*I*) through the specimen [45].

The resistivity *ρ* can be expressed as:

(1) $$\rho =\frac{R_{r}A_{r}}{l}$$

where *l* is the length of the sample at which the voltage is measured and *A* is a cross-section of the specimen. The composite materials' volume has existence the resistivity *ρ* and is resolved by current passing the sample.

(2) $$R_{r}=\frac{\phi }{I}$$

where notation *ϕ* is voltage by loading input (4 V). Furthermore, the electrical conductivity (σ) of the sample can be calculated by the following equation.

(3) $$\sigma =\frac{1}{\rho }$$

## 3 McLachlan theory

McLachlan and the team have significantly enhanced the statistical model by utilizing a generalized effective media equation for systems containing a highly conductive material in a poorly conducting adhesive while taking into account the conductivities of constituent materials. This applies specifically to samples containing adhesive and conductive particles as [46]:

(4) $$\frac{\varphi_{f}\left(\sigma_{f}^{1/t}-\sigma^{1/t}\right)}{\sigma_{f}^{1/t}+A\sigma^{1/t}}+\frac{\left({1-\varphi }_{f}\right)\left(\sigma_{0}^{1/t}-\sigma^{1/t}\right)}{\sigma_{0}^{1/t}+A\sigma^{1/t}}=0$$

where:

(5) $$A=\frac{1-\varphi_{p}}{\varphi_{p}}$$

(6) $$\sigma_{1,2}={\left[-\left(-A\sigma_{0}^{1/t}-\sigma_{f}^{1/t}+A\varphi_{f}\sigma_{0}^{1/t}+\varphi_{f}\sigma_{f}^{1/t}-A\varphi_{f}\sigma_{f}^{1/t}-\varphi_{f}\sigma_{0}^{1/t}\right)\pm \frac{\sqrt{{\left(-A\sigma_{0}^{1/t}-\sigma_{f}^{1/t}+A\varphi_{f}\sigma_{0}^{1/t}+\varphi_{f}\sigma_{f}^{1/t}-A\varphi_{f}\sigma_{f}^{1/t}-\varphi_{f}\sigma_{0}^{1/t}\right)}^{2}-4A\left(\sigma_{0}^{1/t}\sigma_{f}^{1/t}\right)}}{2A}\right]}^{t}$$

The notations above are represented by "*σ*_*0*_″, "*σ*_*f*_", and "*σ*" for electrical conductivities of the adhesives, particles, and composite, respectively. While the particle content is denoted as "*φ*_*f*_", while "*φ*_*p*_" stands for the volume content of the particle at the transition phase. Additionally, “*t*" is a constant parameter. It has been demonstrated by McLachlan [42] that the “*t*" exponent relies on the demagnetisation or depolarization coefficients of the adhesive and particle. However, it's important to note that certain polymers like epoxy exhibit good resistivity, so the term "*σ*_*0*_″ cannot be disregarded for adhesive. Additionally, the particle content of Silver (Ag) in composites is quite low. Therefore, the part (1- *φ*_*f*_) should be considered as 1, simplifying Equation (6) to:

(7) $$\frac{\varphi_{f}\left(\sigma_{f}^{1/t}-\sigma^{1/t}\right)}{\sigma_{f}^{1/t}+A\sigma^{1/t}}+\frac{-1}{A}=0$$

(8) $$\sigma ={\left(\varphi_{f}\sigma_{f}^{1/t}-\frac{\sigma_{f}^{1/t}}{A}\right)}^{t}$$

The modified McLachlan equation provides a precise framework for calculating the performance of electrical conductivity, comprising three elements to simplify the interphase region [40].

(9) $$\frac{\varphi_{f}\left(\sigma_{f}^{1/t}-\sigma^{1/t}\right)}{\sigma_{f}^{1/t}+A\sigma^{1/t}}+\frac{\varphi_{i}\left(\sigma_{i}^{1/t}-\sigma^{1/t}\right)}{\sigma_{i}^{1/t}+A\sigma^{1/t}}+\frac{\left({1-\varphi }_{f}\right)\left(\sigma_{0}^{1/t}-\sigma^{1/t}\right)}{\sigma_{0}^{1/t}+A\sigma^{1/t}}=0$$

(10) $$\sigma_{i}={\left[\frac{\sigma^{1/t}+A\sigma^{1/t}\left\{B\right\}}{1+\frac{\left\{B\right\}}{\varphi_{i}}}\right]}^{t}$$

where:

(11) $$\left\{B\right\}=\left\{\left(1+\varphi_{f}-\varphi_{i}\right)\frac{\sigma_{0}^{1/t}-\sigma^{1/t}}{\sigma_{0}^{1/t}-A\sigma^{1/t}}+\frac{\sigma_{f}^{1/t}-\sigma^{1/t}}{\sigma_{f}^{1/t}-A\sigma^{1/t}}\right\}$$

The notations used are "*φ*_*f*_", "*φ*_*i*_", "*σ*_*i*_", and "*σ*_*f*_", which clearly define the volume content of fiber, the volume content of percolation, and the electrical conductivities for both the composite and the particle. The precise notation is crucial for understanding the relationships within the material properties and their applications. This equation effectively models the region of an interphase zone within a heterogeneous composite model, displaying strong alignment with experimental data in various studies.

## 4 3D finite element model

### 4.1 Formulation on particles dispersion developed

The core particle dispersion technique relies on the computational modelling of particle positions in an iterative system by stochastic motion, allowing particle growth through attractive energy. This technique involves developing the structure of particles in a representative volume element (RVE). The system for particle dispersion employs the following criteria:

- Random generation of seed particles.
- Random launch of particles.
- Calculation of distance (*d*) between seed and launched particles.
- Rejection of particle launch in case of overlap with existing particles, followed by repeating the previous steps.
- If *d* > *d*_*cut*_: in this scenario, when a particle is launched, it unequivocally experiences movement due to the interspace surpassing the attraction effect, resulting in translation around the original seed particle. Here, the parameter “*d*_*cut*_” represents the critical distance over which the attractive van der Waals energy between particles operates. This is a crucial factor to consider in understanding the interactions between particles.
- If *d* < *d*_*cut*_: the launched particle unequivocally gravitates towards and firmly adheres to the original seed particle.
- Finally, the updating of the available coordinate point locations.

### 4.2 Geometry procedure

**Fig. 2:**
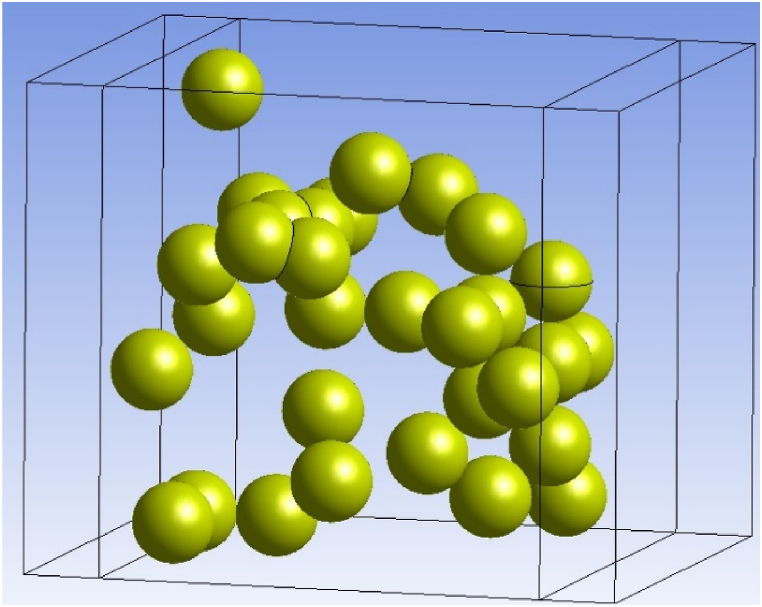
The Ag particles submerged model and coupled by Cu plate.

Unlocking the potential of micro-sized composite, the models feature the use of coupling copper plates to seamlessly facilitate the flow of electric charge, as vividly depicted in Fig. 2. These geometry procedures are created using the commercial CAD software system under ANSYS. The construction of the model involves two crucial steps: generating geometry and particle partitioning. The first step strategy focuses on creating an adhesive block representing the RVE block size and following by situating particles at coordinate positions generated by a developed particle distribution. For the further step, the particles are strategically subdivided within the RVE block matrix model to yield distinct material types.

For the homogeneous particle size, the quantity of particles within the RVE size directly aligns with the filler loading condition. The conditioning of the model should contain all particles for Silver in ranges of 2–8 vol % of volume amount. Further equation illustrates the filler loading impacts the number of Ag particles submerged in the composite model. It's a key factor in our analysis and provides valuable insights into our materials research.

(12) $$Vol.\%=\frac{\left(4/3\right)\pi r^{3}\left(n\right)}{\left(w\cdot h\cdot l\right)}$$

The symbol “*n*" is used to represent the quantity of particle numbers, the next symbols of “*w*,” “*h*,” and “*l*" correspond to the “width”, “height”, and “length” of the RVE model, respectively. The quantity of particle positions generated is directly related to the actual Ag particle content percentage in the RVE model system. As per equation (12), the number of particles with an 80 nm diameter within a medium of 480 nm × 480 nm x 480 nm RVE size is influenced by 8, 16, 24, and 32 particles at the percentage of 2 %, 4 %, 6 %, and 8 % by volume, respectively.

Dependency studies are conducted in simulations for precise and time-efficient results. This analysis involves isolating one parameter while keeping the others constant to confidently observe their impact on the accuracy of the simulation. Convergence results are expected in variable values and the numerical results are shown moving in fixed results. The effectiveness of electrical conductivity in this model is based on the RVE composite block model in terms of variable size during the convergence study. The RVE size was determined from varied particle content to analyses electrical characteristic results. Furthermore, the commercial ANSYS software relies on steady-state conditions to analyze electrical properties.

## 5 Results and discussions

### 5.1 Experimental results

**Fig. 3:**
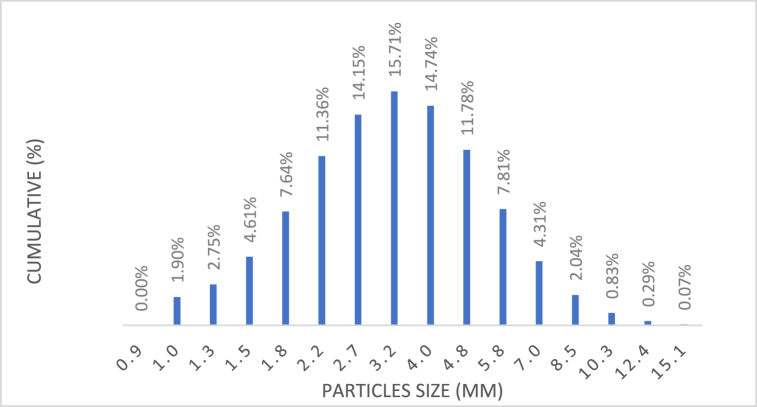
The average diameter particle size of micro.

**Fig. 4:**
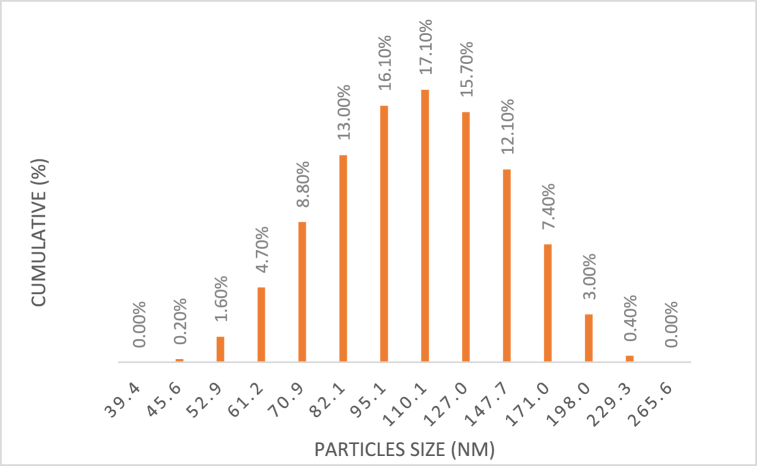
The average diameter particle size of nano.

Fig. 3, Fig. 4 vividly illustrate the intensity relatively varied with all the average diameters of the Silver particles. Notably, both micro- and nano-sized uniform distribution with 15 % and 17 % of all particles within approximately 3 μm and 110 nm of the average diameter, respectively. These findings closely align with the values specified by the suppliers.

**Fig. 5:**
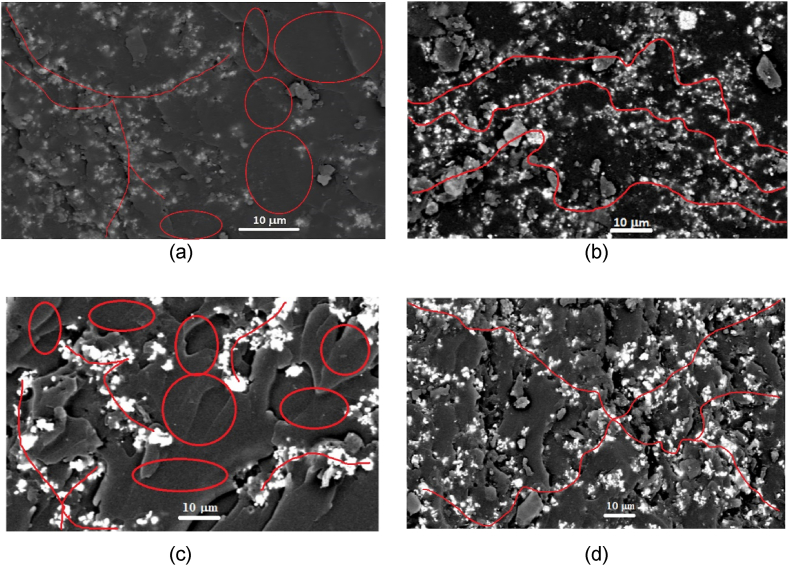
500× magnification of FESEM capture at different silver content: (a) nano-sized at 4 vol%, (b) nano-sized at 6 vol%, (c) micro-sized at 4 vol%, and (d) micro-sized at 6 vol%.

The microstructure of the electrically conductive adhesives (ECAs) observed within the insulator-to-conductor transition provides crucial insights into understanding the formation of conductive networks that enable electrical transport. Fig. 5 presents the microstructure of the electrically conductive adhesives with Silver particle distribution characteristics. The Silver particle distribution is seen within the matrix resin with bright continuous particles' network characteristics. The low electrical conductivity at 4 vol % was a result of insufficient filler content. Specifically, at 4 vol % filler, the Ag particles might be less densely distributed within the epoxy, possibly forming isolated clusters rather than continuous conductive pathways (Fig. 5(a)). The distance between particles can be significantly large, avoiding effective electron tunnelling or conductive percolation networks. This phenomenon can be illustrated by a red circle among particle networks, representing a substantial blocking area. Tunnelling plays a crucial role in the conductivity of materials by allowing electrons from one filler particle to cross the potential barrier and reach an adjacent particle within a specific separation distance. This phenomenon forms a conductive channel between the resins before the particles make direct contact, enhancing electrical connectivity. Notably, tunnelling effects in nano-sized silver (Ag) particles in epoxy conductive adhesives (ECAs) are significant, occurring at distances of around 100 nm, underscoring its importance in advancing electronic applications [47]. Agglomeration creates dense clusters of particles with unclear boundaries, reducing effective surface area and limiting continuous conductive pathways, which increases electrical resistance. Within these agglomerates, some areas may have higher conductivity due to better particle connectivity, leading to uneven electron transport. Voids or insulating materials can also disrupt electron flow.

In Fig. 5(b), the network started to form, leading to an increase in electrical conductivity. This electrical conduction occurs through a path of particles or the Ag particles are more likely to come into contact, forming a continuous conductive network. This is closer to the percolation threshold, where a critical amount of filler is needed to create interconnected paths for electrical flow, thereby increasing electrical conductivity. The fracture surface can reveal details about the mechanical properties of the composite for both micro- and nano-sized. In composites with lower filler content (4 vol%), the epoxy matrix may exhibit smoother fracture surfaces, indicating more polymer dominance (Fig. 5(c). This is clearly illustrated by a red circle within particle networks, which signifies a prominent blocking area and showcases the system's unique capabilities. In contrast, at 6 vol%, the fracture surface may show more particle pull-out and agglomeration, highlighting the interaction between the Ag filler and the matrix (Fig. 5(d). Fig. 5(a) and (b) unequivocally demonstrate that the distribution of silver (Ag) particles was suboptimal, leading to significant agglomeration, as clearly shown in the micrograph. However, the incorporation of nano-sized Ag successfully established a strong particle network necessary for achieving moderate electrical conductivity. Even a small addition of nano-sized Ag was instrumental in creating this effective conductive network. While a consistent amount of filler amplified particle contacts, it also unavoidably increased the potential for agglomeration in the dispersion.

**Fig. 6:**
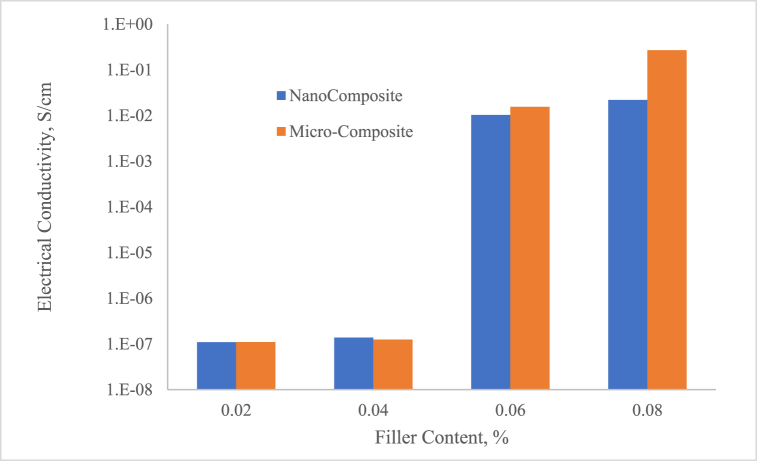
Comparison of electrical conductivity between micro- and nano-sized.

The critical volume fraction of the particle at which a continuous conductive network is formed within the composite matrix. The filler particles are dispersed to form conductive pathways, and the material becomes conducting or exhibits very high conductivity [48]. As the particle content increases and surpasses the percolation threshold, the conductivity increases sharply due to the formation of a continuous network of conductive particles. The key factor determining the percolation threshold is the volume fraction of the conductive filler. Typically, a lower filler content never provides sufficient contact between particles, leading to an insulating matrix. The comparison of the filler volume fraction reaches a critical level (percolation threshold) within micro and nano present in Fig. 6. The phenomenon of both micro- and nano-sized composites has through similar trend in which the conductive paths form throughout the composite, allowing electron transport to begin at 6 % of Silver particles.

The interaction between the filler and the epoxy matrix influences how well the fillers can form conductive networks. If there is a strong bond between the matrix and the filler, it can restrict the mobility of the filler particles and hinder network formation, increasing the percolation threshold. The conductivity of both micro- and nano-sized composite indicate a sudden increase at 6 vol % particle content. The intrinsic electrical conductivity of the composite material directly influences the conductivity of the particle once the percolation threshold is reached. The conductivity of nano-composite reached 1.03 × 10^−2^ S/cm for percolation threshold. The electrically conductive network begins to form, and its precise location because it maximizes particle-particle contact [49]. Uniform dispersion is vital for establishing consistent conductive pathways that significantly enhance electron transport. A homogeneous distribution not only minimizes electron scattering at boundaries and defects but also elevates performance. In composite systems, achieving effective dispersion is essential to surpassing the percolation threshold—the minimum particle concentration required for conductivity. Well-dispersed particles facilitate electron tunnelling between them, dramatically boosting conductivity. Furthermore, a uniform and interconnected network, as evidenced by scanning electron microscopy (SEM), directly translates to superior electrical performance. Employing advanced mechanical processing techniques like sonication, vacuum ovens, and high-speed stirring is crucial for breaking down agglomerates, ensuring maximum efficiency and effectiveness.

The higher electrical conductivity observed in ECAs with micro-sized Silver particles compared to nano-sized particles can be attributed to lower tunnelling resistance and reduced particle agglomeration. Micro-sized particles, with their larger size, form more stable physical contacts, reducing restriction resistance and allowing more efficient conduction. On the other hand, nano-sized particles, while capable of tunnelling, are often hampered by agglomeration and inconsistent spacing, leading to increased resistance and lower overall conductivity [50,51]. One of the major challenges with nano-sized Ag particles is their tendency to agglomerate. Due to the high surface energy of nanoparticles, they tend to cluster together, forming large aggregates. This agglomeration reduces the effective surface area available for conduction and disrupts the formation of a uniform conductive network.

Particles undoubtedly possess a range of structures, including solid spherical and layered forms that are called interphase layers. These structures are essential for effective bonding with polymer morphology, which directly influences the stability and durability of composite materials. Furthermore, they significantly enhance dielectric capabilities, emphasizing their critical role in advanced material applications. The micro-Ag particles in ECAs demonstrate a notably superior electrical conductivity compared to their nano-sized counterparts. This advantage arises from the distinct interphase layers that create contact resistances in the nano-sized system, which detrimentally impact the adhesive's electrical performance. These contact resistances hinder conduction efficiency, meaning the total resistance among particles is essentially the sum of these resistive contacts, directly influencing the overall resistivity. In cases where two particles are connected by a contact surface, each connection behaves like a resistor within the particle network. According to the microstructure image, ECAs achieve conductivity through a robust network of interconnected particles. This model presents the network as a series of linked particles, with each connection adding to the total resistance. A longer particle network chain can significantly increase the resistivity of the composite. Therefore, the electrical transport of nanoparticles should be approached with caution, as it may lead to reduced conduction values and less effective performance overall due to the interphase layer amount. To facilitate this phenomenon in the composite modelling system, individual particles are charged with electrical energy, enabling the transfer of this energy to neighbouring particles through a strategic gaping mechanism. Understanding the maximum distance between particles is essential for effectively addressing the tunnelling effect, which can potentially impact the integrity of the insulating resin. Our study shows that the maximum tunnelling distance between nanoparticles is about 1.6 nm, with electrical conduction analyzed based on this distance, maintaining optimal insulating properties.

### 5.2 McLachlan theory analysis

**Fig. 7:**
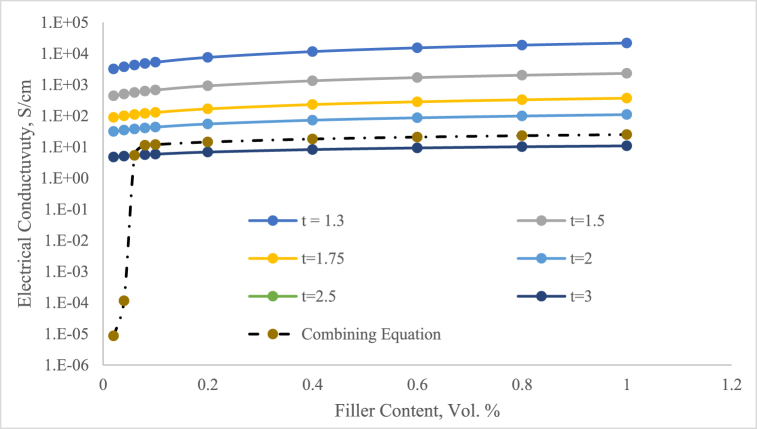
DC electrical conductivity vs. Silver fractions by varied t and combination of the equation.

**Fig. 8:**
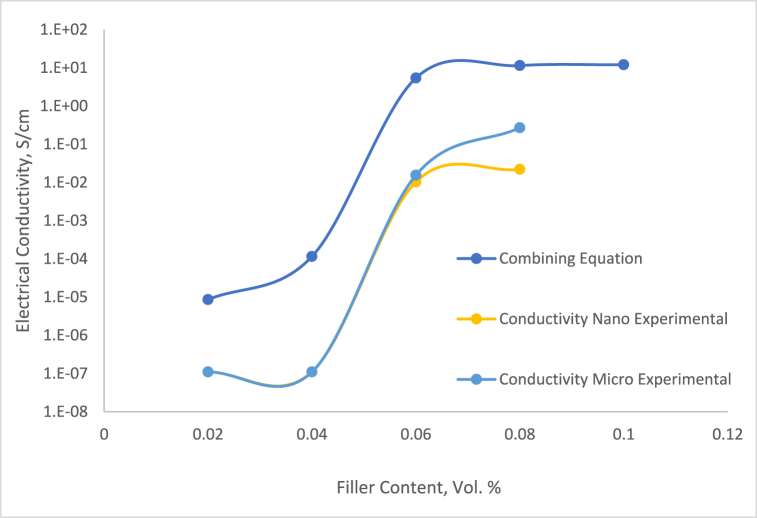
DC electrical conductivity comparison between combining equation and experimental.

The observation of characteristics of the insulator-to-conductor transition phase based on Equation (6) to the particle content in the adhesive system might guide to enhanced conductivity when particles are appropriately volume. The composite's conductivity remains relatively unchanged below the percolation threshold (*ϕp*). Once the particle content changes from insulator to conductor, the electrical properties experience a significant positive effect, reaching a fixed value which characterises the behaviour of fully conducted. The electrical properties of composite refer to the mixture material law where determined through the percolation threshold method. Fig. 7 illustrates the calculation results using Equation (6) for the Silver/epoxy composite. This analysis aimed to validate the equation's accuracy. The discontinuous lines depict the conductivity calculated using Equation (6) and Equation (8) with universal parameter *t* values. Predicted values from percolation theory varied, and by adjusting parameter *t* to achieve a proper set with the experimental results, it was evident that the combination Equation (6) and Equation (8) were indeed valid for Silver content before the percolation threshold (*ϕ* < *ϕp*) (Equation (8)) while Equation (6) for (*ϕ* < *ϕp*) both micro and nano series. The values of the adjustable parameter *t* shown in Table 1, Table 2 demonstrate a strong agreement, surpassing the universal values. These findings corroborate the calculation and study of the McLachlan theory. The Interzone, which separates the condition of phases, has no discernible impact, as the Silver fractions loaded for (*ϕ* < *ϕp*) do not notably affect the conductivity. This has been supported by the data for Silver fractions (*ϕ* > *ϕp*) as illustrated in Fig. 7. In Fig. 7, it is clear that using a general value for the constant *t* never provides an accurate fixed with the two condition phases of the composites. To address this issue, the calculation assumed that the conductivity of these composites was determined using a combination of equations specified in Equation (6) and Equation (8). According to this law, further studies have been done to analyze the effect of filler content in the matrix providing deeper insights into how various filler characteristics. Based on the calculation model, the results obtained from the simulation of Equation (6) and Equation (8) are comparison data with the experimental results in Fig. 8. As seen in this figure, the pattern of the percolation threshold phenomenon is reflected similar pattern in the experimental results. This indicates the important part of the adjustment *t* on conductivity properties characteristics of the threshold system.

**Table 1:** The adjusting parameter *t* to achieve the best fit with the experimental data.

Series *t*	Vol. 2 % (S/cm)	Vol. 4 % (S/cm)	Vol. 6 % (S/cm)	Vol. 8 % (S/cm)
1.3	3.21 × 10^3^	3.76 × 10^3^	4.29 × 10^3^	1.79 × 10^3^
1.5	4.39 × 10^2^	5.04 × 10^2^	5.64 × 10^2^	6.21 × 10^2^
1.7	8.91 × 10^1^	1.00 × 10^1^	1.10 × 10^1^	1.20 × 10^1^
2	3.15 × 10^1^	3.49 × 10^1^	3.80 × 10^1^	4.09 × 10^1^
2.5	9.27	1.01 × 10^1^	1.08 × 10^1^	1.14 × 10^1^
3	4.75	5.09	5.39	5.65

**Table 2:** The adjusting parameter *t* on the combining equation.

Series *t*	Vol. 2 % (S/cm)	Vol. 4 % (S/cm)	Vol. 6 % (S/cm)	Vol. 8 % (S/cm)
8	8.59 × 10^−6^			
6		1.16 × 10^−4^		
2.5			5.39	
2.5				1.14 × 10^1^

**Fig. 9:**
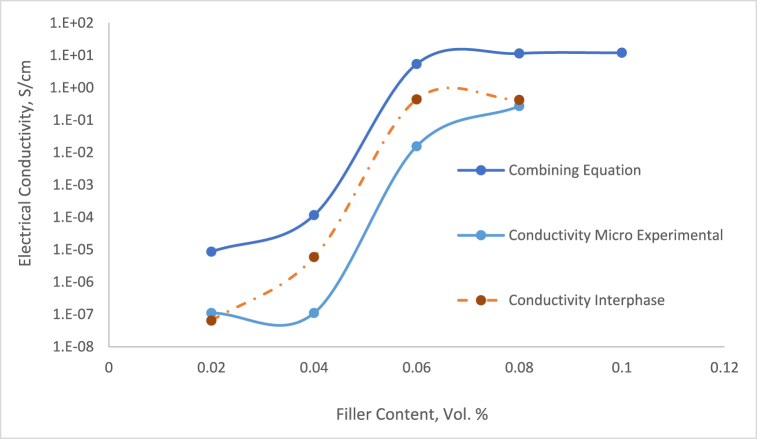
DC electrical conductivity comparison between combining equation and experimental.

The data presented in Fig. 8 clearly shows that using a universal value *t* never precisely represents the fitting of two different material properties. Our study also investigates the impact of the filler/polymer matrix interphase, as detailed in Equation (10), focusing on the interphase region's impact on electric properties. The simulation output of Equation (10) suggests the conductivity of the interphase region, emphasizing its significant role in the electrical path properties of these threshold systems. To analyze the impact of Silver filler on the concentration of the interphase zone, each series was calculated by implementing Equation (10). The outcomes are displayed in Fig. 9, providing a clear illustration of the interphase region values for individually observed sequences of micro-sized composites. Upon comparison, it was evident that the conductivity and pattern of the interphase zone closely aligned with the experimental results compared to combining Equation (6) and Equation (8). McLachlan's theory emphasizes understanding the electrical conduction of electrically conductive adhesives (ECAs) by the absence of the important part of the crucial role of a conductive filler network pattern. Grasping the conduction mechanisms between filler particles is vital for optimizing performance and it is required to comprehend the conduction mechanisms between the filler particles. The characteristics of the particle network can improve our understanding of the conduction pathways within the conductive adhesive.

### 5.3 3D finite element model

**Fig. 10:**
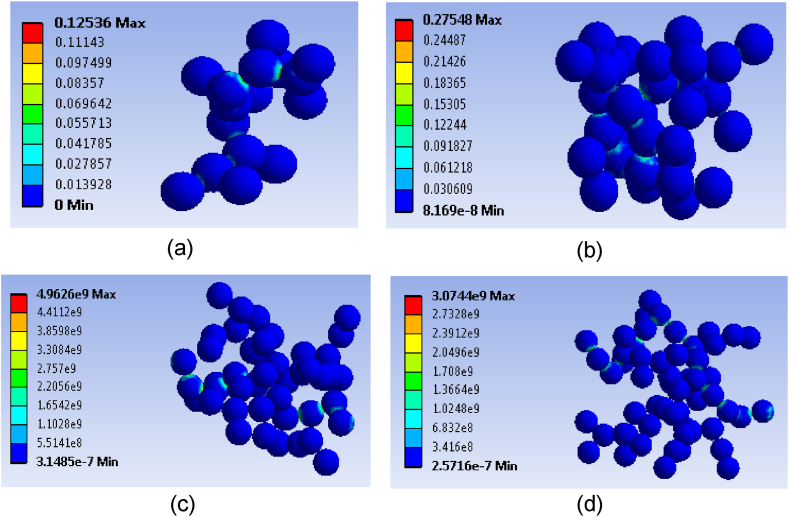
Varied microstructure model at; a) particle content of 2 vol%, b) particle content of 4 vol%, c) particle content of 6 vol % and d) particle content of 8 vol%.

In the microstructure observation, Fig. 10 illustrates the distribution of micro-particles with different filler loadings. The figure displays particle structures at particle content of 2 vol%, until 8 vol % by interval 2 vol % each increment. The analysis revealed two differences in material properties characteristics and structures. In the first place, the characteristic comes within reach of the array and around the model, causing discontinuous array particles to penetrate the model as shown in Fig. 10(a–b). The structural particles develop through particle growth, where particles collide and adhere to the array from any direction. Observing Fig. 10(a–b), it's clear that the microstructure remains insulator regarding lower particle number loadings structures penetrating through the model. This leads to the dominance of agglomeration may not interact effectively with the surrounding particles in the dispersed structures.

The depiction of the second character of particle structure can be seen in Fig. 10(c–d). There are an ample number of particles, enabling the pattern of a continuous path across the model. By adding a sufficient amount of particles, they can come into connecting particles or perform a continuous particle network like an array through the microstructure on any side of the model. The estimated number of particles occurred when the filler content was *V*_*f*_ ≥ 6 vol % (*V*_*f*_ denotes particle content). By including particles one by one, particle walk processes lead to the formation of a network from initial cluster growth in the microstructure model due to van der Waal's law. These continuous particle networks are capable of effectively transporting the electric ion by particle interconnecting.

The distribution of current density characteristics around the particle network was closely examined. It was observed that the contour of the current density level around the particle contact is significantly higher compared to the other side. This phenomenon mirrors the distribution pattern seen in micro-particle cases, where the distribution follows a specific direction to the other side of the model and is arranged in line with the microstructure. This appearance eventuates both before and post the insulator and conductor transition. This analysis revealed a disparity in the level of current density contour before and post the insulator-to-conductor transition phase, particularly at the end of the particle path structure. Fig. 10(a–b) illustrates which is the current density contour becomes visible notably underneath due to the elimination of a difference between the particle microstructure and side, creating difficulty for the insulating state to permeate. Conversely, Fig. 10(c–d) captures the continuous spread of current density distributions across the model, depicted by the higher current density contour.

**Fig. 11:**
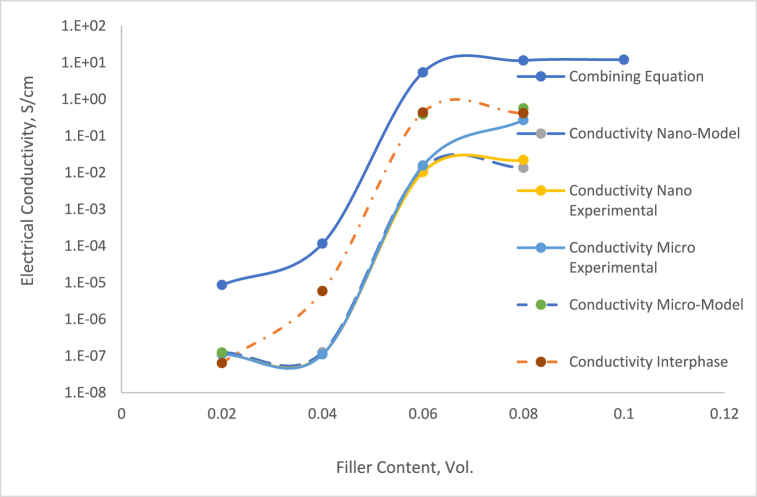
DC electrical conductivity comparison against experimental.

The analysis focuses on the electrical properties before and the post-insulator-to-conductor transition phase, as shown in Fig. 11. Before the threshold, the electrical conductivity demonstrated insulator-like properties, while after the threshold, there was an increase in conductivity. The microstructure model at 2 and 4 vol % of particle content was beneath the crucial content for percolation threshold phenomena for both particle sizes. The graph shows the correlation intervening electrical performance and particle content in the microstructure model. The current density value determines the electrical state before and post the insulator and conductor transition. When the current density was low before meeting the conductive transition, the electrical conductivity acts as an insulator. The slightly huge size eventuates the particles and side of the model hindering the current density penetration across the RVE model. Additionally, 3D finite element models of composites exhibit a higher quality than McLachlan's modified models, showing a significant impact of composite conductivity on percolation threshold patterns. McLachlan's modified theory calculated the highest conductivity due to the absence of an interphase zone, but a modified equation could adjust the conductivity trend in composites. Moreover, the 3D finite elements method successfully approximated the percolation threshold trend to match the experimental results by demonstrating that contact resistance repercussions the conduction value. The final resistance eventuates particles microstructure particle is the total of contact resistance, which influences sum resistivity. When two particles are connected by a contact surface, each interconnecting can be considered a resistor in a particle interconnecting path.

Particle filler plays a crucial role in the structure of electrically conductive adhesives (ECAs) and is essential for understanding electrical conductivity mechanisms. This 3D model study limitation found that focusing on spherical particle shape only needs to be developed by different shapes, particularly flaky forms, and varying particle sizes, it can enhance dispersion and optimize the particle network within ECAs. Additionally, utilizing drag force to influence particle motion during stirring in 3D models can improve dispersion algorithms. Optimizing van der Waals interactions among particles also enhances the formation of strong particle structures and promotes effective growth patterns, driving innovation in ECAs.

## 6 Conclusions

The study proposes a groundbreaking model for predicting the electrical conductivity of composite systems, considering the crucial interphase region eventuates conducting particle and adhesive matrices. By leveraging advanced numerical techniques of combining equations, the model enables the determination of interphase zone volume, intrinsic conductivity, and concentration in composites. In addition, it was evident that the interphase zone significantly influences electrical conductivity, markedly diverging from mixture models that neglect this effect. While the McLachlan-modified model falls slightly short in accuracy compared to 3D finite element method models, adjustments *t* and equations can enhance its alignment with experimental data. The adjusting parameter *t* was varied from 1.3 to 3 to achieve the best fit with the experimental data. Due to the complexities of the McLachlan modified theory, determining the percolation threshold was more challenging compared to the 3D model. The electrical properties at the percolation threshold ranged from 5.39 S/cm to 1.29 S/cm as the parameter *t* was reduced. Moreover, the 3D finite element method model adeptly captures particle motion, mirroring real stirring processes with its portrayal of stochastic motion and translational particle motion on track points simulation. These findings strongly indicate the full formation of particle networks within the structure, with the conductivity closely matching experimental data and revealing an insulator and conductor transition between 4 % and 6 % of particle content.

The Agglomeration Model using the Finite Element Method (FEM) introduces a computationally advanced approach to simulate, analyze, and predict the behaviour of agglomerated and dispersed particles within a matrix. The novelty of using FEM for modelling agglomeration lies in its ability to accurately simulate the spatial distribution of particles and its impact on material properties, particularly conductivity. Several parameters were proposed to make it innovative such as detailed microscopic representation, incorporation of multiphysics phenomena, quantification of interfacial effects, predictive power and dynamic percolation networks. FEM allows for high-resolution modeling of individual particles and clusters, using realistic geometries, sizes, and distributions. Additionally, FEM integrates electrical and physical properties to evaluate how agglomeration influences composite materials. Unlike static models, FEM captures the dynamic evolution of percolation networks, considering real-world factors such as clustering and distribution changes during processing. In material design, FEM provides essential insights into optimizing particle size, shape, and dispersion for desired conductivity. By connecting microstructural features to macroscopic electrical properties, FEM and McLachlan's theory offer powerful tools for developing high-performance materials.

## CRediT authorship contribution statement

**Muhammad Zulkarnain:** Writing – review & editing, Writing – original draft, Visualization, Investigation, Formal analysis, Data curation. **Irianto Harny:** Validation, Methodology. **A.A.M. Damanhuri:** Conceptualization.

## Declaration of competing interest

The authors declare that they have no known competing financial interests or personal relationships that could have appeared to influence the work reported in this paper.

## References

[1] Zhang Li, Li Zefeng, Liu Guiting, Chen Rong, Guo Shaoyun (2023). Enhancement of the electrical and thermal conductivity of epoxy-based composite films through the construction of the multi-scale conductive bridge structure. Compos. Sci. Technol..

[2] Ren Zhenhua, Sun Junbo, Zeng Xiantao, Chen Xi, Wang Yufei, Tang Weichen, Wang Xiangyu (2022). Research on the electrical conductivity and mechanical properties of copper slag multiphase nano-modified electrically conductive cementitious composite. Construct. Build. Mater..

[3] Wolfson H., Elliott G. (1956).

[4] Guo ZhiJin, Lu WenBin, Zhang Yan, Zhou JianPing, Sun DaQian (2023). MXene fillers and silver flakes filled epoxy resin for new hybrid conductive adhesives. Ceram. Int..

[5] Arshad Javid Muhamad, Sadaqat Nazish, Rafique Muhammad, Nabi Ijaz ul, Nadeem Khalid (2021). Synthesis of Ag-Cu based epoxy polymer composite for electrical conductivity. Mater. Today Proc..

[6] Qian Yingjie, Hwang Sosan, Lee Jaewon, Seo Jin Sung, Baeck Sung-Hyeon, Shim Sang Eun (2021). Novel electroless plating of silver nanoparticles on graphene nanoplatelets and its application for highly conductive epoxy composites. J. Ind. Eng. Chem..

[7] Liu Yu Qiang, Zhang Yan, Zhou JianPing, Sun DaQian, Li HongMei (2024). Effect of Ti3C2Tx/Ag/MWCNTs/Ag MXene fillers on the electrical conductivity of Ag-coated Cu conductive adhesives. Ceram. Int..

[8] Zhang Xiao Min, Yang Xiao-Li, Wang Bin (2021). Electrical properties of electrically conductive adhesives from epoxy and silver-coated copper powders after sintering and thermal aging. Int. J. Adhesion Adhes..

[9] Zhang Weiwei, Yao Zhijun, Chen Hongtao (2023). Electrical and mechanical reliability and failure mechanism analysis of electrically conductive adhesives. Microelectron. Reliab..

[10] Chen Yuexi, He Yi, Guo Jiayu, Yang Xiazhen, Guo Bing, Shen Hangyan, Wang Xiaorong (2023). Int. J. Adhesion Adhes..

[11] Sahebi Hamrah Z., Lashgari V.A., Doost Mohammadi M.H., Uner D., Pourabdoli M. (2021). Microstructure, resistivity, and shear strength of electrically conductive adhesives made of silver-coated copper powder. Microelectron. Reliab..

[12] Liu Hao, Liu Jiahao, Wang Shaoan, Jin Zezhu, Zhu Shuyan, Ma Rui, Zhang Weiwei, Wang Jianqiang, Jin Li, Song Chengliang, Zhang Shuye, Chen Hongtao (2022). Effects of silver nano-particles and nano-wires on properties of electrically conductive adhesives. Microelectron. Reliab..

[13] Cui Hui-Wang, Li Dong-Sheng, Fan Qiong, Lai Hua-Xiang (2013). Electrical and mechanical properties of electrically conductive adhesives from epoxy, micro-silver flakes, and nano-hexagonal boron nitride particles after humid and thermal aging. Int. J. Adhesion Adhes..

[14] Zhang Xiaomin (2023). Preparation of silver nanopowders and its application in low temperature electrically conductive adhesive. Microelectron. Reliab..

[15] Lai Yaobin, Zhu Sitian, Li Jian, Zhang Hui, Qi Tao (2023). One-step synthesis of micro-sized flake silver particles as electrically conductive adhesive fillers in printed electronics. J. Ind. Eng. Chem..

[16] Hosseini-Shahisavandi S.M., Zerafat M.M. (2021). Synthesis of carboxylated-silver nanowires: electrical conductivity enhancement of isotropic conductive adhesives and long-term stability in a mixture of solvents. Adv. Powder Technol..

[17] Sukumaran Sheena S., Rekha C.R., Resmi A.N., Jinesh K.B., Gopchandran K.G. (2018). Raman and scanning tunneling spectroscopic investigations on graphene-silver nanocomposites. J. Sci.: Adv. Mater. Devices.

[18] Kainourgios Panagiotis, Tziveleka Leto-Aikaterini, Boukos Nikos, Roussis Vassilios, Charitidis Costas A. (2024). Controlling the growth of uncapped silver nanoparticles on poly methacrylic acid nanospheres: the effect of polymer's free surface on the antimicrobial properties. Colloids Surf., A.

[19] Das Amlan, Chandran Ramkumar, Mallik Archana (2022). Decoration of graphene sheets with silver nanoparticles and their characterization. Mater. Today Proc..

[20] Zhang Li, Li Zefeng, Liu Guiting, Chen Rong, Guo Shaoyun (2023). Enhancement of the electrical and thermal conductivity of epoxy-based composite films through the construction of the multi-scale conductive bridge structure. Compos. Sci. Technol..

[21] Sun Zhijian, Li Jiaxiong, Yu Michael, Kathaperumal Mohanalingam, Wong Ching-Ping (2022). A review of the thermal conductivity of silver-epoxy nanocomposites as encapsulation material for packaging applications. Chem. Eng. J..

[22] Sunil J., Dhayanithi Pooja M., Ginil R., Alex S.N., Ajith Pravin A. (2021). Thermal conductivity and dynamic viscosity of aqueous-silver nanoparticle dispersion. Mater. Today Proc..

[23] Al-Gburi Ahmed Jamal Abdullah, Ismail Mohd Muzafar, Mohammed Naba Jasim, Buragohain Akash, Alhassoon Khaled (2024). Electrical conductivity and morphological observation of hybrid filler: silver-graphene oxide nanocomposites for wearable antenna. Opt. Mater..

[24] Lee Wondu, Park Sang Duck, Kim Jihoon, Park Dabin, Whang Dongmok, Kim Jooheon (2024). Development of copper-boron nitride core-shell structured fillers and surface treatment for thermally conductive composites. J. Alloys Compd..

[25] Chen Qingguo, Yang Kailun, Feng Yu, Liang Liang, Chi Minghe, Zhang Zhonghua, Chen Xuesong (2024). Recent advances in thermal-conductive insulating polymer composites with various fillers. Composites, Part A.

[26] Teixeira S.C., Gomes N.O., de Oliveira T.V., Fortes-Da-Silva P., de F.F Soares N., Raymundo-PereiraP A. (2023). Review and Perspectives of sustainable, biodegradable, ecofriendly and flexible electronic devices and (Bio)sensors. Biosens. Bioelectron..

[27] amjid E.T., Najafi P., Khalili M.A., ShokouhnejadN, Karimi M., Sepahdoost N. (2024). Review of sustainable, eco-friendly, and conductive polymer nanocomposites for electronic and thermal applications: current status and future prospects. Discover Nano.

[28] Dias O.A.T., Konar S., Leao A.L., Yang W., Tjong J., Sain M. (2020). Current state of applications of nanocellulose in flexible energy and electronic devices. Front. Chem..

[29] Hegde V.N., Pradeep T.M., Manju V.V., Sandhya N.C. (2024). MgO nanofiller reinforced biodegradable, flexible, tunable energy gap HPMC polymer composites for eco-friendly electronic applications. Mater. Sci. Eng. B.

[30] Hadi Zahra, Yeganeh Jafar Khademzadeh, Munir Muhammad Tajammal, Zare Yasser, Rhee Kyong Yop (2024). An innovative model for electrical conductivity of MXene polymer nanocomposites by interphase and tunneling characteristics. Composites, Part A.

[31] Zare Yasser, Munir Muhammad Tajammal, Rhee Kyong Yop, Park Soo-Jin (2024). Effects of a deficient interface, tunneling size and interphase depth on the percolation inception, percentage of graphene in the nets and conductivity of nanocomposites. Diam. Relat. Mater..

[32] Zare Yasser, Rhee Kyong Yop (2020). Expression of characteristic tunneling distance to control the electrical conductivity of carbon nanotubes-reinforced nanocomposites. J. Mater. Res. Technol..

[33] PayandehpeymanM J., Khamehchi MazaheriM. (2020). Prediction of electrical conductivity of polymer-graphene nanocomposites by developing an analytical model considering interphase, tunneling and geometry effects. Compos. Commun..

[34] Haghgoo M., Ansari R., Hassanzadeh-Aghdam M.K., Nankali M. (2019). Analytical formulation for electrical conductivity and percolation threshold of epoxy multiscale nanocomposites reinforced with chopped carbon fibers and wavy carbon nanotubes considering tunneling resistivity. Composites, Part A.

[35] Kim Do-Won, Hyuk Lima Jae, Yu Jaesang (2019). Efficient prediction of the electrical conductivity and percolation threshold of nanocomposite containing spherical particles with three-dimensional random representative volume elements by random filler removal. Composites, Part B.

[36] Xu Wenxiang, Wang Wei, Guo Weiqi, Jia Mingkun, Jiao Yang (2023). Soft interphase volume fraction of composites containing arbitrarily shaped mono−/poly-disperse fillers: theoretical and numerical investigations. Powder Technol..

[37] Mortazavi B., Baniassadi M., Bardon J., Ahzi S. (2013). Modeling of two-phase random composite materials by finite element, Mori-Tanaka and strong contrast methods. Compos. Part B Eng..

[38] Odegard G.M., Clancy T.C., Gates T.S. (2005). Modeling of the mechanical properties of nanoparticle/polymer composites. Polymers.

[39] Brosseau Christian (2024). Modeling the interface between phases in dense polymer-carbon black nanoparticle composites by dielectric spectroscopy: where are we now and what are the opportunities?. Macromol. Theory Simul..

[40] Aribou N., Nioua Y., El Hasnaoui M., Achour M.E., Costa L.C. (2020). Effect of filler/matrix interphase boundaries on the DC electrical conductivity of two- and three-dimensional composites. J. Optoelectron. Adv. Mater..

[41] Zare Yasser, Rhee Kyong Yop (2019). Simplification and development of McLachlan model for electrical conductivity of polymer carbon nanotubes nanocomposites assuming the networking of interphase regions. Composites, Part B.

[42] Lutza Melanie P., Zimmerman Robert W. (2016). Effect of the interphase zone on the conductivity or diffusivity of a particulate composite using Maxwell's homogenization method. Int. J. Eng. Sci..

[43] Lipatov Yu S., Kosyanchuk L.F., Yarovaya &N.V. (2006). In situ polymer nanocomposites : effect of nanoparticles on the interfacial region in situ polymer nanocomposites : effect of nanoparticles on the interfacial region. Compos. Interfac..

[44] Zare Yasser, Rhee Kyong Yop, Hui David (2017). Influences of nanoparticles aggregation/agglomeration on the interfacial/interphase and tensile properties of nanocomposites. Composites, Part B.

[45] Liu YuQiang, Zhang Yan, Zhou JianPing, Sun DaQian, Li HongMei (2023). Effect of Ti_3_C_2_Tx/Ag MXene fillers on the electrical conductivity of Ag-coated Cu conductive adhesives. Ceram. Int..

[46] McLachlan D.S., Blaszkiewicz M., Newnham R.E. (1990). Electrical resistivity of composites. J. Am. Ceram. Soc..

[47] Wu H.P., Wu X.J., Ge M.Y., Zhang G.O., Wang Y.W., Jiang J.Z. (2006). High conductivity of isotropic conductive adhesives filled with silver nanowires. Int. J. Adhesion Adhes..

[48] Weber M., Kamal M.R. (1997). Estimation of the volume resistivity of electrically conductive composites. Polym. Compos..

[49] Wang Qian, Zhang Shuye, Liu Guiming, Lin Tiesong, He Peng (2020). The mixture of silver nanowires and nanosilver-coated copper micronflakes for electrically conductive adhesives to achieve high electrical conductivity with low percolation threshold. J. Alloys Compd..

[50] Wua H.P., Liua J.F., Wu X.J., Gea M.Y., Wang Y.W., Zhanga,c G.Q., Jianga J.Z. (2006). High conductivity of isotropic conductive adhesives filled with silver nanowires. Int. J. Adhesion Adhes..

[51] Qu M., Xie Z., Liu S., Zhang J., Peng S., Li Z., Lin C., Nilsson F. (2023). Electric resistance of elastic strain sensors—fundamental mechanisms and experimental validation. J. Nanomater..

